# Manic Episode after Ventricular-Peritoneal Shunt Replacement in a Patient with Radiation-Induced Hydrocephalus: The Role of Lifetime Subthreshold Bipolar Features

**DOI:** 10.1155/2014/953728

**Published:** 2014-01-12

**Authors:** Antonio Callari, Valentina Mantua, Mario Miniati, Antonella Benvenuti, Mauro Mauri, Liliana Dell'Osso

**Affiliations:** ^1^Section of Psychiatry, Department of Clinical and Experimental Medicine, University of Pisa, Via Roma 57, 56100, Italy; ^2^European Assessment Office, Italian Medicines Agency (AIFA) Via del Tritone 181, 00187 Rome, Italy

## Abstract

We present a case report of a woman hospitalized for a ventricular-peritoneal shunting replacement, who developed a manic episode with psychotic symptoms after hydrocephalus resolution. We have no knowledge of cases of manic episodes due to hydrocephalus resolution by ventricular-peritoneal shunt replacement, although previous case reports have suggested that hydrocephalus might induce rapid-onset affective episodes or mood cycles. The patient's history revealed the lifetime presence of signs and features belonging to the subthreshold bipolar spectrum, in absence of previous full-blown episodes of a bipolar disorder. Our hypothesis is that such lifetime sub-threshold bipolar features represented precursors of the subsequent full-blown manic episode, triggered by an upregulated binding of striatum D2 receptors after the ventricular-peritoneal shunt replacement.

## 1. Introduction

Neuropsychiatric manifestations, although uncommon, have been described in association with hydrocephalus (a condition caused by obstruction in drainage, decreased absorption or excess production of cerebrospinal fluid). Normal Pressure Hydrocephalus (NPH) may present with its classic progressive triad (abnormal gait, ataxic/apractic dementia and urinary incontinence) [1], as paranoid psychosis [2, 3] or as a mood disorder, such as depressive episodes, manic episodes [4–6], or mood cycling [7, 8]. As far as we know, there is no knowledge of mood episodes with the onset after hydrocephalus resolution. We present the case of a woman hospitalized for a ventricular-peritoneal shunt replacement after a radiation-induced hydrocephalus, who developed a manic episode with psychotic symptoms when hydrocephalus remitted. The patient was treated with antipsychotics and completely recovered. Apparently no previous mood episodes had been manifested in her lifetime history. However, the information collected during hospitalization revealed a lifetime history characterized by several indicators of a bipolar spectrum diathesis. We hypothesized that the ventricular-peritoneal shunting might be associated with an increased dopaminergic tone and that this condition was able to trigger the manic episode in a patient with a lifetime history of subthreshold bipolar manifestations. This hypothesis derived from two recent positron emission tomography studies that found a reduced binding of striatum D2 receptors in normal pressure hydrocephalus [9] and an upregulated binding after the ventricular-peritoneal shunting [10]. Moreover, both studies showed that changes in striatum D2 receptors were correlated with changes in emotional and cognitive functions.

## 2. Case Presentation 

Mrs. A. is a 37-year-old woman who was admitted to the Neurosurgery Clinic of the University of Pisa. An MRI brain scan revealed a hydrocephalus for a shunt obstruction. The patient had already been treated with surgery and radiotherapy at the age of 30. At that age, during the postpartum period, the patient developed tingling and muscle weakness with abnormal gait. She complained also of a transient demoralization, with loss of energy and apathy, impaired concentration and generalized anxiety. The concomitant severe muscle weakness prompted her general practitioner to prescribe a brain scan. The CT scan revealed the presence of a thalamic glioblastoma for which Mrs. A. was successfully treated with surgery and radiotherapy. A mild left hemi paresis persisted localized in the left hand and leg that improved in few months with physiotherapy. After this event, she was euthymic for 2,5 years, and the mood/anxiety symptoms presented during the postpartum period were considered as secondary to the glioblastoma. Two and a half years after surgery-radiotherapy treatment, she suffered from psychomotor retardation, energy loss, drowsiness, and impaired concentration and memory. In a few days she became comatose. An MRI scan revealed a post-radiation hydrocephalus that was successfully treated with a ventricular-peritoneal shunt placement. She maintained a good physical condition for 5 years. Then, Mrs. A. presented again with psychomotor retardation, apathy and loss of energy. The MRI brain scan revealed a recurrence of the hydrocephalus for a shunt obstruction. The patient was hospitalized and successfully treated with a ventricular-peritoneal shunting replacement. Two days after the shunt replacement, MRI scan revealed the resolution of the hydrocephalus, with a marked reduction of the right lateral ventricles and the re-expansion of the underlying and adjacent brain structures. She was discharged from the Neurosurgery Clinic. Two days after dismissal, she unexpectedly became hyper-talkative and irritable. She developed a subtotal insomnia, with grandiosity, flight of ideas, and persecutory delusions. Two days after, she was referred to the Psychiatric Clinic of the University of Pisa. The neurological assessment was negative (except for a mild residual left hemiparesis localized in the left hand and leg); blood investigations were unremarkable. A neurosurgical clinical consultation and a CT brain scan confirmed the successful ventricular-peritoneal shunting replacement. The patient met the diagnostic criteria for a manic episode with psychotic symptoms. She was treated with typical and atypical antipsychotics (Haloperidol 3 mg/daily and Quetiapine 600 mg/daily) combined with the antiepileptic drugs already prescribed by neurosurgeons (Phenytoin 200 mg/daily and Gabapentin 600 mg/daily). The episode remitted in 7 days, and Mrs. A. was discharged.

## 3. Discussion 

Apparently, the lifetime history of the patient was negative from a psychiatric point of view. Mrs. A. did not report previous depressive or manic/mixed, hypomanic episodes. Nonetheless, in a more accurate lifetime assessment subthreshold manifestations belonging to the realm of bipolar spectrum emerged, which revealed a number of signs and symptoms that had been completely overlooked.

- Mrs. A. describes herself as a “*sensitive and serious person*,” usually introverted, frequently disappointed in herself, precise, with an “*unstable temperament inherited from the mother.*” Actually, Mrs. A's. mother suffered from a major depressive episode when she was about 42.
- At the age of 21, Mrs. A. had a two-month period characterized by a subjective change in energy levels. The patient started a diet and suddenly became hyperactive, with a weight loss of about 8 kg. She felt more assertive, mentally sharp, brilliant and clever, more confident, extroverted, and sociable, with constantly high levels of energy, decreased need for sleep, and increased self-esteem. Mrs. A. did not take any medication; the described condition spontaneously diminished within two months; after that period, the patient became again “*an average person*.”
- Mrs. A. suffered with marked demoralization during the postpartum period, with mood instability, trouble with thinking or concentrating, a need for more sleep, difficulties in making even minor decisions, loss of interest and pleasure concomitantly with high levels of anxiety. As already described, she reported also severe muscle weakness and a marked loss of energy that leaded to a CT scan revealing the presence of a thalamic glioblastoma. As a consequence, the signs and symptoms described in the postpartum period were, at that time, diagnosed as being secondary to the medical condition.

A number of case reports suggest that hydrocephalus can trigger mood episodes and mood cycles [1, 8]. Chronic normal pressure hydrocephalus is usually associated with mood and cognitive dysregulations while the rapid evolution of hydrocephalus towards a comatose state may provide little time for the appearance of psychiatric symptoms. Mrs. A. manifested a first episode of hydrocephalus characterized by depressive-like symptoms that evolved rapidly towards a coma and a second episode that was treated with the shunt replacement. After the second hydrocephalus resolution by the shunting the patient suffered from a manic phase. Interestingly, two recent positron emission tomography (PET) studies demonstrated that the binding of striatum D2 receptors was reduced in normal pressure hydrocephalus [9] and upregulated after ventricular-peritoneal shunting [10]. So we might hypothesize that an increased dopaminergic tone may be associated with development of manic symptoms, as in our case, after ventricular-peritoneal shunting. Nonetheless, it is unclear why Mrs. A. did not develop a manic phase after the first hydrocephalus, when the clinical course was different and the hydrocephalus, as already noticed, evolved rapidly towards a coma. We argue that patients who develop symptoms of a manic episode in comorbidity with the ventricular-peritoneal shunting might be a “*special population*” of subjects with a lifetime diathesis for bipolar spectrum manifestations that were not detected or reported by clinicians or patients. In our case, a family loading for mood disorders, cyclothymic temperamental traits, at least one episode of mild hypomania, and the presence of depressive signs and symptoms in the postpartum period were present even if they were completely overlooked, since the full-blown manic episode was triggered.

There are several reasons for the low rate of detection of subthreshold lifetime manic manifestations among patients with hydrocephalus. These include the lack of subjective suffering, enhanced productivity, egosyntonicity, and clinicians' tendency to subsume persistent or even alternating symptoms among the “*unwanted consequences*” secondary to an “*important neurological condition*.” The central diagnostic importance placed on hydrocephalus might distract clinicians from paying attention to other more subtle but clinically meaningful symptoms, such as changes in energy, neurovegetative symptoms and distorted cognitions that are strictly related to a soft bipolar diathesis. Given that, we propose that special attention should be devoted to the systematic detection of lifetime mild mood manifestations, especially among patients who have neurological conditions such as hydrocephalus that have been found to mimic a bipolar disorder.
